# The Self-Simulational Theory of temporal extension

**DOI:** 10.1093/nc/niad015

**Published:** 2023-06-19

**Authors:** Jan Erik Bellingrath

**Affiliations:** Center for Mind and Cognition, Ruhr-Universität Bochum, Universitätsstraße 150, Bochum, Nord-Rhein-Westfalen, Germany

**Keywords:** temporal now, self-model, computational modelling

## Abstract

Subjective experience is experience in time. Unfolding in a continuous river of moments, our experience, however, consists not only in the changing phenomenological content per se but, further, in additional retrodiction and prospection of the moments that immediately preceded and followed it. It is in this way that William James’s ‘specious present’ presents itself as extending between the past and future. While the phenomenology of temporality always happens, in normal waking states, to *someone*, and the notions of self-representation and temporal experience have continuously been associated with each other, there has not yet been an explicit account of their relationship. In this paper, the emergence of the subjective experience of temporal extension will be conceived of as arising out of a difference-relation between counterfactual and actual self-representations. After presenting the proposed relationship on both a conceptual level and a formalized and neuronally realistic level of description using information theory, convergent empirical evidence from general findings about temporal experience and inference, altered states of consciousness, and mental illness is examined. The Self-Simulational Theory of temporal extension is able to explain systematic variations in the subjectively experienced length of the temporal Now across numerous domains and holds potentially wide implications for the neuroscience of consciousness, as well as for a deeper understanding of different forms of mental illness.

## Introduction: the temporal flow

‘…Hours fly by like minutes, and minutes are oppressively slow, as if they were centuries.’ (Rovelli 2019)

The subjective experience of time seems all-prevalent. As does the subjective experience of change. However, even though time as a physical phenomenon might be exhaustively reducible to change, the same cannot be said about the subjective experience of time (Kent and Wittmann 2021). Being embedded in a temporal river, an extended Now, William James’s ‘specious present’ (James et al. 1890), the experience of the subjective present is not simply the experience of change, or change of change, etc., but rather is supplemented by continuous retrodiction and prospection (Kent and Wittmann 2021). It is by retrodicting the moments that immediately preceded, and by prospecting or predicting the moments that are assumed to be subsequently likely to follow, that temporal experience unfolds in an extended, continuous stream. This counterfactual extension in subjective temporality, with ‘counterfactual’ referring to prospections/retrodictions of the world represented as non-actual, far from being vague and uncertain by necessity, can in principle be conceived of by accounting for precisely those processes in ourselves that are responsible for that counterfactual embedding. One possibility for these embedding-instantiating processes is the representation of actual and counterfactual states of selfhood. Indeed, phenomenologically speaking, it is true that, in the normal waking state, there is not only the temporal river itself but always *someone* flowing in that very river (for a naturalized of selfhood, see Metzinger 2003). This notion of systematic covariance between expressions of selfhood and temporal experience, with the covariance being too close for a coincidence to be likely, has its historical roots in the west, amongst others, in Edmund Husserl and Maurice Merleau-Ponty (for new editions, see Merleau-Ponty 2013, Husserl 2019). Indeed, over the last years and in diverse disciplines, evidence for a close relationship between the two phenomena has been accumulated. In psychology, over and above numerous connections between the two phenomena (Droit-Volet et al. 2013, Wittmann 2015), diverse psychopathological conditions are characterized by co-occurring changes in self- and time-related inferences (see e.g. Kent et al. 2021; see also Vatakis and Allman, 2015). Neuroimaging research has, equally, from diverse areas of study, time and again identified significant associations between self-representations and temporality (see e.g. Wittmann et al. 2010, Lloyd 2012; for a meta-analysis, see Nani et al. 2019). Furthermore, the insular cortex, the primary interoceptive cortex integrating bodily, i.e. self-referential, signals, has not only been identified as being centrally involved in time perception in the sub- and supra-second range (see e.g. Lewis and Miall 2003; for two recent meta-analyses, see Naghibi et al. 2023, Mondok and Wiener, 2022) but has even been postulated to be the single-most essential neural basis of time perception as such (Craig 2009; see also Kosillo and Smith 2010). However, even though this close association between self-referential processing and time-related processing has been referenced across levels of description, methods, tasks, and theoretical perspectives, a theory explicitly relating the two in the sense of one being emergent from the other has been lacking. Accordingly, in the following, a specific mechanism will be proposed, by which the subjective experience of temporal extension embedded in that elusively hovering temporal Now is an automatically emergent phenomenon resultant from a specific type of self-referential processing, self-simulation. The emergent temporal structure is conceptually distinct from both time as a physical phenomenon and other time-keeping mechanisms of the brain, as will be discussed. After conceptually describing, and mathematically formalizing the relationship on a neuronal level, converging evidence will be presented.

## Self-simulation as the foundation of temporal extension

Conscious experience unfolds in time. Indeed, it may seem that if temporality were subtracted from our experience, consciousness itself would vanish (Metzinger 2003). Whether true or not, temporality is a seemingly ever-present phenomenon; and estimates for the *width* of the temporal moment range back over the millennia, with the Buddhist *Treasury of Abhidharma*, e.g., estimating there to be 65 temporal moments in the time it takes a man to snap his fingers; with the length of the temporal Now thereby becoming about 1/65 of a second (Thompson 2014). Self-representations and counterfactual self-simulations are, as well, an extremely prevalent phenomenon in our perception. Counterfactual-self-simulation, or the ‘self-as-counterfactual’ as opposed to self-representation or the ‘self-as-actual’, refers to simulations of in principle possible, yet not currently actual, states of the internal systemic model that is the Self (see Metzinger 2003, for similar notions, as well as for a conceptually more fine-grained analysis of components of selfhood, such as the bodily- or narrative-self, that the current theory abstracts away from). In the following, these slightly unusual terms ‘self-as-actual’ and ‘self-as-counterfactual’ will be used, in order to free the notions of possible connotations.

While counterfactual-self-simulations can take many different forms, such as in mind-wandering or dreaming, they are of central-most importance for human beings. Simulating what might happen to oneself and what could be done about it makes it possible for human beings to simulate possible outcomes of a given future situation, enabling him or her to align his or her behaviour with the (self-)simulation of a given situation, judged to be most desirable (see e.g. Buckner and Carroll 2007). The importance of counterfactual-self-simulations for choosing appropriate behaviour among possible future versions of ourselves stands in accord with their prevalence. While studies have estimated human beings to spend about half of their waking hours mind-wandering (Killingsworth and Gilbert 2010), mind-wandering is not the only form of counterfactual-self-simulation. Counterfactual-self-simulations express themselves in dreams and hypnagogic and hypnopompic imagery (the visuo-auditory phenomena while falling asleep and waking up, respectively), as well as in theory-of-mind-related capabilities such as empathy, perspective taking, and the inference of intentions. Furthermore, the terms counterfactual-self-simulation, or the self-as-counterfactual, as they are intended to be used here, not only denote phenomena occurring on a hierarchically high, or classically conceived, ‘cognitive’ level of processing but are also explicitly intended to denote lower-level self-referential sensorimotor processes, such as the counterfactual prediction to soon place a limb in a certain position, with the limb of the self-as-actual moving forward to close the gap (see Friston 2009, Clark 2015).

A common denominator of most counterfactual-self-simulations is the fact that there always has to be, by necessity, a process constituting an automatically occurring comparison between the self-as-actual and the self-as-counterfactual. For example, a counterfactual-self-simulation arising in mind-wandering of a desired and hoped-for or far-fetched and feared future-self has to be compared to the actual-self. If there was no process instantiating an emergent comparison of the counterfactual and the actual, the counterfactual-self could not be represented as hoped for or feared in the first place since it is precisely the deviation from the current situation and systemic state by the self-as-counterfactual that encodes its motivational valence (see Clark 2015), as intuitively evidenced by the fact that a given outcome of a situation differs in its inter-individually attributed valence.

A given comparison between the self-as-counterfactual and the self-as-actual is therefore characterized by a specific degree of dissimilarity of the contents being related. The degree of dissimilarity between the self-models corresponds, anthropomorphisingly speaking, precisely to the ‘change that would have to happen in order for (a part of) me to become that other (part of) me’.

The central claim of the Self-Simulational Theory (SST) of temporal extension is that the automatically emergent difference-relation between the self-as-actual and the self-as-counterfactual corresponds to the subjective experience of the extension of time; the subjective extension of time is equivalent to the lived experience of the ‘length of your way to another version of yourself’.

This follows from the fact that the self-as-counterfactual, while not being integrated with the bodily self in the three-dimensional world ‘here, now’ directly, is in principle reachable in it. By means of the non-actual-self being a counterfactual possibility that ‘just might occur’ as an embedded and embodied phenomenon in three dimensions, and by means of it being characterized by a specific distance (dissimilarity metric) of how far away it is from being actual, it extends in a dimension that is not one of the bodily three dimensions but subsumes them as its actuality-constituting anchor. The representation of a dimension that is not three dimensional in and of itself, but whose counterfactually represented contents might in principle become three-dimensional actuality, is simply what a representation of temporality conceptually entails. In this way extends every simulated self as a counterfactual possibility to a degree in subjective time, that is equivalent to the amount of change that would have to occur for it to be identical to the self-as-actual. Temporal experience, the extension in the retrodicted and prospected temporal river, is identical to the amount of change a self-model has to undergo in order to transform into another, counterfactually, yet concretely, represented self-model. Temporal experience is an extension in an emergent dimension of self-simulational counterfactuality.

It is in this way that individual instances of counterfactual-self-simulations are varying along a dimension of counterfactuality, with the automatically emergent dissimilarity metric specifying the distance between the self-as-actual and the self-as-counterfactual being the ‘change that would have to occur for me to transform into that other me’. The dimensional dissimilarity metric itself is automatically emergent for the reason outlined earlier: the self-as-counterfactual could not be associated with its motivational valence if it were not in a comparison-constituting relation to the self-as-actual; stars shine most brightly at night and the fall is greater close to the sun. The automatically emergent difference-metric between the self-as-actual and the self-as-counterfactual, the ‘change that I have to undergo to transform into that other me’, constitutes the experience of temporal extension; the experience of temporality is extension in a dimension of self-simulational counterfactuality.

Self-simulations, furthermore, are always characterized by a specific extent of representational detail. This extent of representational detail, of both actual- and counterfactual-self-simulations, however, limits the in-principle possible difference between them. If the counterfactual- (or actual-)self is not multifaceted, complex and detailed in the first place, then there are not many individual degrees of freedom the self-as-actual (or -counterfactual) could be in principle different from. While the distance relation between self-models, therefore, is bounded by the extent of their representational detail, the amount of extension in counterfactual-self-space varies—different self-models will result in different extents of dissimilarity between them. This varying distance between counterfactual- and actual-self-simulations, this varying extent in an automatically emergent dimension of self-simulational counterfactuality, corresponds to subjectively experienced temporal contraction and dilation, differing degrees of how long a moment subjectively feels. Furthermore, as a consequence of the level of the representational detail constituting an in-principle-bound of the extent of dissimilarity, the reason why a thought about one’s long-gone childhood-self or a thought about one’s far-away retirement does not directly lead to the perception of staring eternity itself in its eye, is simply that the possibility for representing very distant self-models and live circumstances vanishes with their degree of representational distance. A long-gone or far-away version of a given self-as-counterfactual is simply too abstract to be enriched with a lot of representational detail; the in-principle length of the ‘way I would have to undergo to become that other me’ is limited by the extent of representational detail. In the following, the difference-relation between the self-as-actual and the self-as-counterfactual, temporal extension, will be formally expressed on a neuronal level via tools from information theory.

## Information-theoretic formalization on the level of the neuronal ensemble

Even though Shannon (1956), the originator of information theory, has warned against the over-inflated use of the concepts, information theory has a central position in many scientific fields, the neuroscience of consciousness amongst them (see e.g. Hoel 2017, Albantakis et al. 2022; for general information on information-theoretic neuroscience, see Dimitrov et al. 2011; specifically relating to the following formalization, see Gupta and Bahmer 2019).

In the current context of formalizing the automatically emergent distance relation between self-simulata, the extent of counterfactuality can ‘in principle’ be modelled on several different levels of spatiotemporal grain, presupposing different conceptual frameworks. The relationship can, e.g., be formalized on the level of (not necessarily spatially proximate) individual neuronal ensembles. That is, we can presume that, although we might not precisely know where the individual neurons are, that there are some such, possibly distributed, neurons, conjointly forming a neuronal ensemble, that are functionally underlying the self-as-actual and the self-as-counterfactual, respectively. It is also, to give another example, possible to formalize the self-simulational difference-metric over activation distributions of neuronal populations—which in turn are given a functional reading (see e.g. Schöner and Spencer 2016). Population coding locates the relevant level of description for perception, action, and cognition largely on the level of neuronal populations (see e.g. Schöner 2020), and it has increased over the last decades in popularity. Although there are, over and above these two examples, several other descriptive levels on which the relationship may be modelled, the present formalization will concern itself, motivated mainly by the possibility for shorter and possibly simpler expression, with the descriptive level of the neuronal ensemble. Therefore, the self-simulational metric will in the following be formalized on the descriptive level of (possibly distributed) neuronal ensembles.

The information-theoretic entropy, *H*, denotes the average information-theoretic ‘surprise’, after observing all outcomes of *m* neurons, which are either active (spike) or inactive (no-spike), with *p (X)* denoting the probability function governing the activity of the respective neuron.


$$H\left( X \right)\, = \, - \mathop \sum \limits_{i = 1}^m {p_i}log\left( {p\left( X \right)} \right)$$

The mutual information of two variables, on the other hand, is a general measure of the strength of the association between those two variables (Stone 2015). Specifically, given two variables *X* and *Y*, the mutual information *I(X; Y*) is the average reduction in uncertainty about one of the variables, *X* or *Y*, when knowing the value of the other. For the application of mutual information in neural circuits, the probability distributions of the random variables *X* and *Y*, two separate pools of *m* and *n* neurons, represent the probability distributions of the binary states of the neurons as either active or inactive, with *x* and *y* representing the individual neurons in the respective neuronal pool. The mutual information between *X* and *Y* is defined to be the following, where *H(X)* denotes the entropy and *I*(*X; Y*) the mutual information:


$$\,I\left( {X;Y} \right) = H\left( X \right) - H\left( {X{\rm{|}}Y} \right)\; = \mathop \sum \limits_{x \in X}^m \mathop \sum \limits_{y \in Y}^n p\left( {{x_i},{y_i}} \right)lo{g_2}\frac{{p\left( {{x_i},{y_i}} \right)}}{{p\left( {{x_i}} \right)p\left( {{y_i}} \right)}}$$

If the states (active or inactive) of the neurons in the two neuronal pools are completely independent of each other, the mutual information is zero, while, on the other hand, the mutual information is maximized when knowing about the complete state of one neuronal ensemble completely specifies the state of the other neuronal ensemble (for further details, see Gupta and Bahmer 2019). Put into other words, and verbally relating mutual information to the information-theoretic entropy *H*, the mutual information between two variables is identical to the difference of the independent uncertainty about one variable minus the uncertainty of that very variable, after accounting for the information being contained in the other variable. Initial uncertainty minus remaining uncertainty is the information explained. However, while the quantity itself is symmetrical, the quantity does not require an actual reciprocal connection between the two neuronal circuits (Gupta and Bahmer 2019).

The extent of dissimilarity between the self-as-actual and the self-as-counterfactual can be expressed accordingly. The extent of dissimilarity between two self-simulations, $D\left( {{S_A};{S_C}} \right)$, is the extent to which the self-as-actual is, by and of itself, unable to intrinsically informationally (mutual information) specify the neuronal state corresponding to the self-as-counterfactual.


$$D\left( {{S_A};{S_C}} \right) = \,H\left( {{S_C}{\rm{|}}{S_A}} \right)\; = \,H\left( {{S_C}} \right)\, - \,I\left( {{S_C};{S_A}} \right)$$

The extent of the dissimilarity between the self-simulations, the ‘change I would have to undergo to transform into that other me’, corresponds to the intrinsically remaining information-theoretic entropy of the self-as-counterfactual from the conditioned perspective of the self-as-actual. Essentially, the distance between the self-as-actual and the self-as-counterfactual decreases incrementally with further progression on the path/dissimilarity reduction of the ‘change I have to undergo to become that other me’, until it dissolves in the actualization of the self-as-counterfactual entirely. It is in this way that the width of the temporal Now extends to a varying degree in counterfactuality, and the James’s ‘specious present’ emerges (James et al. 1890). The width of the experienced temporal Now, following SST, is neither constant nor reducible to externally tracked durations, such as by clocks. In the words of Rovelli (2019), experientially it holds that ‘…Hours fly by like minutes, and minutes are oppressively slow, as if they were centuries’.

## Systematic empirical variations in experiences of temporal extension

Has your smartphone ever fallen down from some height? Have you ever perceived yourself in danger, perhaps in falling yourself? So common, so intuitive, is an increased dragging, an increased slowing down of the experience of temporality in these situations, that it would seem almost redundant to cite evidence for this all-too-prevalent phenomenon. It seems to be the case, indeed, that time subjectively slows down (extends) when negative stimuli are expected. Duration is overestimated when painful stimuli are anticipated (Ogden et al. 2015), when fearful videos are seen (Pollatos et al. 2014), or even simply when a visual stimulus is approaching, which, caused by its ambiguous nature, just might be a threat to the organism (Wittmann et al. 2010). Classically, negative stimuli get processed more deeply (Baumeister et al. 2001; see also Alves et al. 2017), with the difference between the anticipating self-as-actual and the unfortunate self-as-counterfactual increasing in virtue of this increased processing depth. Put in other words: the difference between the self-as-actual and the negatively-to-be-affected self-as-counterfactual increases by virtue of the deeper processing for negative stimuli; and it is this increase in counterfactual distance that corresponds to the perceived dragging, or slowing, of subjective time experience quoted earlier. If the self-simulational distance increases, subjective time extends, and we overestimate duration. Furthermore, not only are negative stimuli by virtue of demanding more processing depth associated with temporal extension, but the same holds equally true for stimuli of larger magnitude and more complex or intense stimuli (Eagleman 2008, Van Wassenhove et al. 2008). On the other hand, repeated and high-probability stimuli — stimuli, that, by their already-expected nature, demand less processing, and an accordingly smaller counterfactual distance, contract time accordingly (Eagleman 2008, Van Wassenhove et al. 2008). If the self-simulational distance decreases, subjective time contracts, and we underestimate duration. Even at its very extremes, such as in potentially life-threatening accidents, a situation in which the self-as-counterfactual is arguably the most deviant from the self-as-actual, by virtue of being predicted as being dead or potentially so, does the same pattern hold true: ‘time stood still’ (Noyes and Kletti 1976, 21). On the other end of the spectrum of the extremity of experience, time also drags when we are waiting or bored (Zakay 2014), with empirical evidence also showing dispositionally boredom-prone individuals as relatively over-estimating duration in the seconds-to-minutes range (Danckert and Allman 2005). With nothing on our hands but episodes of mind-wandering whose prevalence starkly increases in precisely those situations in which no external task is present (Smallwood and Schooler 2015), the difference between the selves-as-counterfactually created during such episodes and the self-as-actual increases, leading, as in the other cases, to a subjective extension of temporality. If the self-simulational distance, the distance between the self-as-actual and the self-as-counterfactual, increases (on average over an episode), subjective time extends (slows down), and we overestimate duration. Furthermore, self-regulation, the implication of a counterfactual-self that has to be reached via effortful action, is associated with a feeling of prolonged duration (Vohs and Schmeichel 2003), a feeling of prolonged duration created by a representation of ‘all those effortful things I still have to do to finally reach that other me’. Individuals with depression and anxiety, furthermore, show a systematic bias towards an overestimation of duration, alongside an experienced expansion of temporality (Bschor et al. 2004). As elaborated earlier, negative stimuli, such as internal self-simulations in anxious or depressed thought episodes, are characterized by an increased processing depth (Baumeister et al. 2001), and it is this increased (average) difference between the self-as-actual and the self-as-counterfactual that SST takes to explain the subjective experience of time slowing down, with temporal overestimations being resultant. In attention deficit hyperactivity disorder, a disorder characterized by a comparatively high degree of impulsivity, a recent meta-analysis has identified affected individuals to have a tendency towards temporal overestimation (Zheng et al. 2022; see also Rubia et al. 2009, Wittmann et al. 2011). This temporal overestimation, conceptually identical to the examples given earlier, can be explained by conceiving of impulsivity as resultant from a comparatively high degree of difference between the self-as-actual and the self-as-counterfactual, with impulsive acts by the individual being the hasty attempt to bridge this perceived gap, which, by virtue of it being larger than in unaffected individuals, leads to a relative overestimation of the ‘change the self-as-actual would have to undergo to become the self-as-counterfactual’, the extension of subjective temporality. It has been shown that stimulus-dependent individuals, as would be expected by SST via a similar argument, also tend towards temporal overestimation (Wittmann et al. 2007). Another set of systematic variations of temporal experience, that is potentially explainable by SST, is constituted by altered temporal experiences in depersonalization disorder, autism, and schizophrenia. The three disorders have been described, respectively, as constituting central disorders of self-related processing [depersonalization disorder (see e.g. Ciaunica et al. 2022), autism (see e.g. Gillespie‐Smith et al. 2018; though see also Nijhof and Bird 2019), and schizophrenia (see e.g. Sass and Parnas 2003, Sass et al. 2018)] and are furthermore closely associated with altered temporal experience [depersonlization disorder (Simeon et al. 2007, 2008), autism (Casassus et al. 2019), and schizophrenia (Thoenes and Oberfeld 2017, Ueda et al. 2018)]. Although this general theme could be understood in terms of SST, specific evidence is potentially relative to both the conceptualizations of the respective illnesses (see e.g. Guloksuz and van Os 2018) and models of the underlying mechanisms, and the complexity of the illnesses and their relation to temporal experience generally demand a more thorough treatment than can be given here; this constitutes a valuable avenue of further research. With regard to non-pathological, yet altered, states of experience, the flow state, a state characterized by self-forgetting absorption in a task that presents just the right amount of challenge (Nakamura and Csikszentmihalyi 2014), not only has been linked with changes in the experience of both selfhood and temporal experience but is, even more so, in questionnaires that measure its occurrence, explicitly defined with regard to the self-absorbed aspect that constitutes the experience (e.g. Rheinberg et al. 2019). Temporal duration is underestimated in the flow state, as evidenced by both qualitative reports and experimental findings (Csikszentmihalyi 1991, Hancock et al. 2019). Indeed, the intuitive association of the self-absorbed flow state with altered (fastened) temporal experience seems to hold on a preconceptual level to such a degree that participants spontaneously reverse-infer having been in the flow state, given that they were (falsely) informed about much time having passed (Christandl et al. 2018). To have a lot of experimental time passed is to have been self-forgotten. Similarly, advanced meditators report a co-occurring dissolution of the experience of time and selfhood in diverse meditative traditions and exercise variants, culminating in a state of ‘pure experience’, a state characterized by a drastic decrease in both self-referential and temporal processing (Gamma and Metzinger 2021, see also Laukkonen and Slagter 2021). If, in deep states of meditation, there are less alternate self-simulations, being characterized by a specific extent of ‘change that I would have to undergo in order to become that other me’, there is less temporal experience since it is precisely the extent of change (difference-relation) in a counterfactual dimension of self-simulational possibilities that constitute time experience in the first place.

On a more general level, episodic memory, centred around self-simulations of our life embedded in its broader socio-cultural and global context, is what orients in time. Episodic memory informs us about actions we have done, their consequences, our resultant hopes and fears, and their relationship to the broader context in the changing stream of our experience. If SST is correct, however, and conceptually independent of issues of whether time as a physical phenomenon is fundamental or not, our subjective experience of our phenomenal extension in time is resultant from that very process that anchors us in a sequential manner in physical time. Traces of the past, permitted in the macroscopic world by its thermodynamic entropy gradient (Rovelli 2022), include our memory traces. Self-simulational processes extending to memory traces, which underlie or constrain our counterfactual-self-simulations, more generally, can only develop in a world in which the very persistence of memory traces is physically possible (Buonomano and Rovelli 2021, Rovelli 2022). So, while the temporal extension in the phenomenal Now is from one side conceptually orthogonal to the physics of time (physical time might pass without self-simulational processes ever existing), it is nevertheless deeply grounded in the way the physics of our universe operates, and constrains, the cognitive possibilities of living beings.

## The relationship of SST to models of time-keeping and the neuroscience of consciousness more broadly

The SST of temporal extension is a theory whose epistemic goal is constituted by the phenomenal extension of temporal experience. While it is possible to assume that our judgements about a differing extent of temporal extension, such as when we are bored or having fun and time goes devastatingly slow or way-to-quickly, are systematic misattributions that reify the notion of the length of the experienced Now beyond its underlying reality, to date, there has not been a unifying account that is able to explain why this should necessarily be so and how we could account for the systematic variations in perceived temporal extension under this pre-conception. While, under SST, temporal experience unfolds as an emergent difference-metric in a space of self-simulational possibilities, this self-relational way of measuring duration is far from being the only way of encoding temporal intervals. Time-keeping, the encoding of duration or intervals of physical time, unfolds in the brain across many levels of spatiotemporal scale and across various different mechanisms, extending from the day-spanning circadian clock (Richards and Gumz 2013) to specific mechanisms responsible for sub- and supra-second intervals (see e.g. Buonomano 2007, Paton and Buonomano 2018). For example, in the sensorimotor domain, models of neuronal population clocks describe high-dimensional neuronal population dynamics whose time evolution intrinsically encodes differing durations (Karmarkar and Buonomano 2007, Buonomano and Laje 2011). Neuronal population clocks are both theoretically parsimonious and empirically plausible, and their primary explanatory target is a neuronal encoding of duration as opposed to the width of temporal experience (see Kent and Wittmann 2021). While there are numerous ways in which the brain keeps time and tracks duration, with domains varying from the day-spanning circadian clock (Richards and Gumz 2013) to short temporal windows underlying sensory integration (for a review of temporal integration processes, see Wittmann 2011), these mechanisms of duration keeping are in principle, especially in relation to shorter time scales, coextensive with the relationship SST proposes; they might simply be descriptions on different levels of description. The epistemic goal of SST, however, extends over and above an encoding of duration. While it is implied by SST that the brain uses the automatically emergent self-simulational difference-metric to encode duration, and this encoding of duration is indeed what deviates from externally tracked clock time in cases of altered temporal experience, such as when we lose ourselves in the flow or are confronted with too much self-simulational activity in episodes of boredom, the epistemic goal of SST consists of the temporal window, the length of the Now, itself. Motivationally necessitated comparisons between what is (the self-as-actual) and what ought (or ought not, the self-as-counterfactual) corresponds to a specific amount of extension in the space of self-simulational possibilities. Extension in an emergent dimension of (self-simulational) counterfactuality, a dimension that subsumes the three-dimensional (bodily part of the) self-as-actual as its representational anchor, is, by its four-dimensional representational nature, what extension in phenomenal temporality conceptually presupposes and hence the proposed mechanism. By anchoring temporality in self-simulational comparisons, and ultimately the bodily self, SST describes temporal width and experience as fundamentally embodied and predicts extension in the temporal river in every cognizing system equipped with sufficiently similar self-modelling [for a naturalized account of selfhood, see Metzinger (2003), whose Self-Model Theory of Subjectivity presents one way of understanding ‘sufficiently similar’ in this context].

*If* SST presents an adequate model of the emergence of the temporal Now, this could possibly have important implications for the neuroscience of consciousness. SST presents an information-compressed explanation of large regions of phenomenal state space. The emergent self-simulational temporal Now itself, however, might not only constitute a characteristic and essential feature of human consciousness but extend to non-human animals as well as artificial systems. Furthermore, SST might help adjudicate between, and theoretically advance, different theories of consciousness, relating especially to the integration of ‘temporal consciousness’ (Kent and Wittmann 2021).

## Conclusion

Consciousness unfolds in time. Living in an extended present, a continuous flow, it is this extension of the Now, however, which *a priori* demands explanation — given that time as a physical phenomenon just ‘is’ and does not extend. The SST of temporal extension starts from the assumption that self-simulational activity is not only central to brain function, but virtually all-prevalent. Self-evidencing dynamics (Howhy 2016, Friston 2018) of what might be done, what has been done, how one could move, how one did move, and how one should have moved instead are simply what free-energy-minimizing generative models are entailed to do to ensure the long-term avoidance of their entropic dissolution (Friston 2009). Counterfactual-self-simulations, however, must be compared to actual-self-simulations; otherwise, their implications for goal-directed action could not be evaluated; what ought is representationally always in relation to what is. The perceived ‘length of the way to another version of yourself’, temporal extension, is the natural dissimilarity metric resultant from a comparison anchored on the three-dimensional bodily self-as-actual, being subsumed by a fourth representational dimension of self-simulational counterfactuality. While the difference-metric itself has been formalized with information-theoretic entropy, this specific mathematical formalization (as well as the level of the description on which it is applied, as elaborated earlier) is only one possibility. Further theoretical work connecting the present formalization, as well as possible variations thereof, to (formalized) models of general characteristics of cognitive systems (e.g. Friston 2009, Schöner and Spencer 2016, Beggs 2022) might lead to a deeper understanding/refinement of the theory, paving the way towards a deeper understanding of temporal variations in various mental illnesses, as well as to a deeper understanding of the phenomenal-state-space itself.

‘Time is the substance I am made of. Time is a river which sweeps me along, but I am the river.’ (Borges 2007)
